# Mulberry Fruit Extract Ameliorates Nonalcoholic Fatty Liver Disease (NAFLD) through Inhibition of Mitochondrial Oxidative Stress in Rats

**DOI:** 10.1155/2018/8165716

**Published:** 2018-12-13

**Authors:** Dong Kwon Yang, Dong-Gyu Jo

**Affiliations:** ^1^Department of Veterinary Pharmacology and Toxicology, College of Veterinary Medicine, Chonbuk National University, Iksan, Jeollabuk-do 54596, Republic of Korea; ^2^School of Pharmacy, Sungkyunkwan University, Suwon 16419, Republic of Korea; ^3^Biomedical Institute for Convergence, Sungkyunkwan University, Suwon 16419, Republic of Korea

## Abstract

Mulberry is known to have pharmacological effects against cholesterol, obesity, and dyslipidemia. Many studies have revealed that mulberry leaf possesses hepatoprotective properties against nonalcoholic fatty liver disease (NAFLD); however, mulberry fruit is less studied in this context. Therefore, this study aimed to investigate the preventive effects of mulberry fruit against high fat diet- (HFD-) induced NAFLD. To evaluate the effects of mulberry fruit on NAFLD, two doses of mulberry fruit ethanol extracts [MB, 100, and 200 mg/kg BW (body weight)] were given to HFD-fed rats for 10 weeks. MB dramatically prevented liver damage as shown by biochemical analysis of the liver injury markers, alanine transaminase, and aspartate transaminase. MB treatment significantly inhibited the increased levels of total cholesterol, triacylglycerol, and low-density lipoprotein-cholesterol but restored the level of high-density lipoprotein-cholesterol in HFD-fed rats. Notably, histological analysis of liver tissues demonstrated that MB substantially ameliorated lipid accumulation. Expression of cholesterol-regulating genes was also suppressed by MB treatment. For its underlying mechanisms, MB suppressed hepatic reactive oxygen species (ROS) overproduction and mitochondrial oxidative stress in HFD-fed rats. MB potentially protects liver tissue against NAFLD by inhibition of mitochondrial oxidative stress, suggesting its possible use as a therapeutic agent for treatment of NAFLD.

## 1. Introduction

Non-alcoholic fatty liver disease (NAFLD) is the most common chronic liver disease worldwide [1, 2]. Obesity caused by overnutrition is associated with several chronic diseases, including metabolic syndrome, fatty liver, and cardiovascular diseases [3–5]. NAFLD, defined as excessive fat accumulation in the liver, can progress to nonalcoholic steatohepatitis (NASH), cirrhosis, and hepatocellular carcinoma [6–9]. Therefore, excessive lipid accumulation is a key pathological process in the early stages of NAFLD before progression to liver fibrosis and inflammation [10–12].

Oxidative stress resulting from imbalance between pro-oxidants and antioxidants induces many pathological events in the liver. Oxidative stress is a crucial mechanism involved in the pathogenesis of NAFLD [13]. Mitochondria are required for energy production via oxidative phosphorylation of glucose and lipid in the liver [14]. Particularly, because mitochondria are vulnerable to reactive oxygen species (ROS) due to oxidative stress, maintenance of mitochondrial function is important for cell survival under oxidative stress [15]. Indeed, considerable evidence suggests that mitochondrial dysfunction is directly related to the pathogenesis of NAFLD [16]. Mitochondrial dysfunction leads to overproduction of ROS that inhibit the lipid metabolism and cause for lipid peroxidation and hepatocyte apoptosis [17]. In addition, it can reduce the activity of the mitochondrial respiratory chain (MRC) to further aggravate adenosine triphosphate (ATP) synthesis and increase oxidative stress. Previous studies have also shown that intermittent ROS change hepatic cell redox conditions and activate kinases that are sensitive to redox reactions, resulting in hepatic steatosis [18, 19]. Therefore, regulation of mitochondrial oxidative stress by antioxidants may be a novel strategy for the treatment of NAFLD.

Phenolic compounds, flavonoids, and anthocyanins, which are physiologically active substances in fruits and vegetables, have antioxidant effects and are now recognized as a potential strategy for preventing obesity and obesity-related metabolic syndrome [20–22]. Mulberry extract is rich in phenols, flavonoids, anthocyanins, and many other antioxidants and is used in food and pharmaceutical compounds due to its pharmacological effect [23, 24]. Previous studies have reported that mulberry leaf possessed ameliorative effects against dyslipidemia and lipid accumulation in high fat diet- (HFD-) fed mice [25, 26].

A number of pharmacological studies of mulberry fruit extract have been done on cell-type model [27–30]. However, the pharmacological properties of mulberry fruit have been less studied as well as those of mulberry leaf against NAFLD.

In this study, we determined whether mulberry fruit can prevent NAFLD induced by a high-fat diet in rats, especially focusing on the prevention of oxidative stress and restoration of mitochondrial functions as underlying mechanisms.

## 2. Materials and Methods

### 2.1. Ethics Statement

All animal procedures for this study were approved by the Institutional Animal Care and Use Committee of Chonbuk National University Laboratory Animal Center (CBNU2016-67), and all efforts were made to minimize animal suffering.

### 2.2. Preparation of Mulberry Extract

Mulberry fruit was purchased from a local market in Sujuchon, Yecheon, Korea. The fruit was dried in an incubator at 60°C and powdered in an electric blender. The dried powder was then extracted in 70% ethanol at room temperature for 24 h. The extracts were filtered, evaporated in a rotary vacuum evaporator, and lyophilized. The powder was kept at 4°C for further experiments.

### 2.3. Animal Study Design and High-Fat Diet Feeding

Male Sprague-Dawley rats weighing 240-260 g were obtained from OrientBio Co. (Sungnam, Korea). Rats were maintained on a 12 h:12 h light:dark cycle in cages and acclimated under laboratory conditions for at least one week before experiments. The control group was fed a standard diet, whereas the HFD groups were fed a calorie-rich diet containing 1% cholesterol, 18% lipid (lard), 40% sucrose, 1% AIN-93G vitamins, and 19% casein, with the same fiber and minerals as the control standard diet, for 10 weeks. For mulberry extract (MB)-treated groups, MB was administered in doses of 100 or 200 mg/kg BW (body weight). Rats were divided into four groups (n=10 in each group): (1) control group treated with distilled water (DW); (2) HFD-fed group; (3) HFD-fed + 100 mg/kg BW of MB; (4) HFD-fed + 200 mg/kg BW of MB. Rats were administrated either DW or indicated dose of MB by oral gavage. For sacrifice of rats, they were anesthetized with CO_2_ inhalation to minimize suffering.

### 2.4. High-Performance Liquid Chromatography (HPLC) Analysis

Anthocyanins, including cyanidin 3-glucoside (C3-G) and cyanidin 3-rutin (3C-R), were measured on a Kromasil 100-5 C18 column (4.6 mm x 250 mm) using an HPLC system (Thermo Electron Co., Beverly, MA, USA). A gradient elution was carried out with solvent A (formic acid:water = 10:90,* v*/*v*) and solvent B (acetonitrile:methanol:formic acid:water = 22.5:22.5:1.5:48.5,* v*/*v*) for analysis of C3-G and C3-R. The flow rate was 1.0 mL/min. Absorption spectra of anthocyanins were recorded from 240 nm to 600 nm with an inline PDA detector.

### 2.5. Biochemical Analysis

Serum total cholesterol (TC), triglycerides (TG), aspartate aminotransferase (AST), alanine aminotransferase (ALT) (Asan Pharmaceutical Co., Seoul, South Korea), and high-density lipoprotein (HDL)/low-density lipoprotein- (LDL-) cholesterol (Biovision, Milpitas, CA, USA) levels were measured using commercial enzymatic kits. For quantification of liver TG, the hepatic lipids were extracted using a binary solution of chloroform/methanol solution (2:1,* v*/*v*). After obtaining supernatant by centrifugation at 12,000 x g for 10 min, the TG level was measured using a commercial TG Assay Kit from Asan Pharmaceutical Co.

### 2.6. Quantitative Real-Time PCR (qRT-PCR)

After harvesting liver tissues, total RNA was extracted using TRIZOL reagent (Invitrogen, Grand Island, NY, USA) according to the manufacturer's instructions. For qRT-PCR analysis, reverse transcription PCR was performed on total RNA with Superscript III reverse transcriptase (Thermo Fisher Scientific, Waltham, MA, USA) and oligo-(dT) primer (Promega, Madison, WI, USA). Quantitative PCR was performed with the iQ SYBR Green PCR Supermix in the DNA Engine Opticon real-time system (Bio-Rad Laboratories Inc., Hercules, CA, USA). Primer sequences were as follows: SREBP-1c forward: 5′- GACGACGGAGCCATGGATT-3′, SREBP-1c reverse: 5′-GGGAAGTCACTGTCTTGGTTGTT-3′; ACC forward: 5′-TGAGGAGGACCGCATTT ATC-3′, ACC reverse: 5′- GAAGCTTCCTTCGTGACCAG-3′; FAS forward: 5′-TCCCAGGTCTTGCCGTGC-3′, FAS reverse: 5′-GCGGATGCCTAGGATGTGTGC-3′: SCD-1 forward: 5′-CACCCAGCTGTCAAAGAGAAGG-3′, and SCD-1 reverse: 5′-AGGACGATATCCGAAGAGGTGG-3′.

### 2.7. Western Blot Analysis

Proteins were isolated from liver tissues using RIPA buffer containing a protease inhibitor cocktail (Roche Diagnostics, Basel, Switzerland). Proteins were then separated by 7% SDS-PAGE and transferred to PVDF membranes (Millipore, Billerica, MA, USA). After 2 h blocking with 5% non-fat milk, the blot was probed with primary antibodies against NADPH oxidase 4 (NOX-4, Abcam, Cambridge, MA, USA) and *β*-actin (Santa Cruz Biotechnology, Santa Cruz, CA, USA). Next, the membranes were incubated with horseradish peroxidase- (HRP-) conjugated secondary antibodies (Cell Signaling Technology, Danvers, MA, USA) for 1 h at room temperature and detected by enhanced chemiluminescence.

### 2.8. Dihydroethidium (DHE) and MitoSOX Staining

Intracellular ROS production was quantified by measuring the fluorescence intensities of DHE (Thermo Fisher Scientific) and MitoSOX (Thermo Fisher Scientific) as ROS indicators. Frozen liver tissues were sectioned with 10 *μ*m thickness. Tissue sections were then washed in phosphate-buffered saline (PBS) and incubated with 20 *μ*M DHE and 5*μ*M MitoSOX at 37°C for 30 min. The DHE and MitoSOX stained cell areas were quantified using a Zeiss LSM 510 META confocal microscope (Carl Zeiss, Jena, Germany).

### 2.9. Measurement of Hepatic Lipid Peroxidation

The protein contents of lipid peroxidation products, including 4-hydroxynonenal (4-HNE) and malondialdehyde (MDA), were measured by commercial colorimetric assay using the LPO-586 kit (Oxis International Inc., Portland, OR, USA). Briefly, liver tissues were homogenized in 20 mM phosphate buffer, pH 7.4, containing 0.5 mM butylated hydroxytoluene (BHT) to prevent oxidation. The homogenates were then centrifuged at 3,000 x g for 10 min at 4°C. After a color reaction was performed for 5 min at room temperature, absorbance was measured at 450 nm using a spectrophotometer (Spectra Max M5; Molecular Devices, Sunnyvale, CA, USA).

### 2.10. Measurement of Hepatic SOD Activity

The hepatic SOD activity was determined by a colorimetric method using a SOD activity assay kit (Biovision). Briefly, the liver tissue was homogenized in 0.1M Tris-Cl, pH 7.4, containing 0.5% Triton X-100, 5 mM *β*-mercaptoethanol, and 0.1 mg/ml phenylmethylsulfonyl fluoride (PMSF) and then centrifuged at 14,000 x g for 10 min at 4°C. After a color reaction for 20 min at 37°C, the absorbance was measured at 450 nm using a spectrophotometer (Molecular Devices).

### 2.11. NADPH Oxidase Activity

NADPH oxidase activity was measured by the lucigenin-enhanced chemiluminescence method as previously described [31]. Briefly, 50 *μ*g of membrane proteins fractionated from the frozen livers was added to Krebs-Ringer buffer containing 1 mM EGTA, 150 mM sucrose, 5 *μ*M lucigenin, and 100 *μ*M NADPH, pH 7.0. Photon emission in terms of relative light units was measured by the luminometer every 30 s for 5 min. There was no measurable activity in the absence of NADPH. The NADPH oxidase activity was expressed as relative chemiluminescence (light) units (RLU)/mg protein. Protein contents were measured by the BCA protein assay reagent (Thermo Fisher Scientific).

### 2.12. Determination of Activities of Mitochondrial Complexes I and III

Mitochondrial complex I and III activities were measured using a MitoTox™ OXPHOS Complex I and III Activity Kit (BioVision) according to the manufacturer's instructions. Complex I activity was detected using a spectrophotometer by measuring the decrease in NADH absorbance at 600 nm. Complex III activity was monitored at 550 nm based on the reduction of cytochrome c in the presence of reduced decylubiquinone.

### 2.13. Measurement of Total ATP Content in Rat Liver

Liver samples were homogenized in perchloric acid and centrifuged at 15,000 g for 2 min. The supernatant was collected, and 30 *μ*l was added to a 96-well plate and brought up to 50 *μ*l with ATP assay buffer. ATP measurement was performed according to the manufacturer's instructions using the ATP colorimetric/fluorescence assay kit (BioVision).

### 2.14. Histological and Immunohistochemical Analyses

Liver tissue was fixed in 10% buffered formaldehyde and embedded in paraffin. Sections of 4 *μ*m thickness were cut and stained with hematoxylin and eosin (H&E) and Oil-red-O. For immunohistochemistry, the sections were dewaxed and rehydrated with serial alcohol gradients. After dewaxing, the slides were incubated overnight at 4°C with 4-HNE antibody (Millipore Corporation). The slides were then washed three times with PBS, and endogenous peroxidase was blocked with 3% H_2_O_2_ in absolute methanol for 30 min at room temperature. Next, the slides were incubated with EnVision+ System-HRP (DAKO, Glostrup, Denmark) for 45 min at room temperature. Finally, the reaction products were stained with diaminobenzidine (DAB), counterstained with Mayer's hematoxylin, and mounted with Eukitt mounting medium after drying. Images were acquired and analyzed with ImageJ software.

### 2.15. Statistical Analysis

All data are expressed as the mean ± SEM. Data were analyzed using a one-way analysis of variance (ANOVA) with the Tukey post hoc test using Prism 5.03 (GraphPad Software Inc., San Diego, CA, USA).* P* < 0.05 was considered as statistically significant.

## 3. Results

### 3.1. Compositional Analysis of Anthocyanins in MB

Previous studies have reported that mulberry extract contains two main anthocyanins, cyanidin 3-glucoside and cyanidin 3-rutin [32, 33]. Therefore, HPLC analysis was performed to determine the composition of C3-G and C3-R as standard components for validation of MB. The results showed that MB contained 15.3 and 5.3%, respectively (Table 1).

### 3.2. Effects of MB on Hepatic Steatosis in HFD-Induced NAFLD

**Figure 1:**
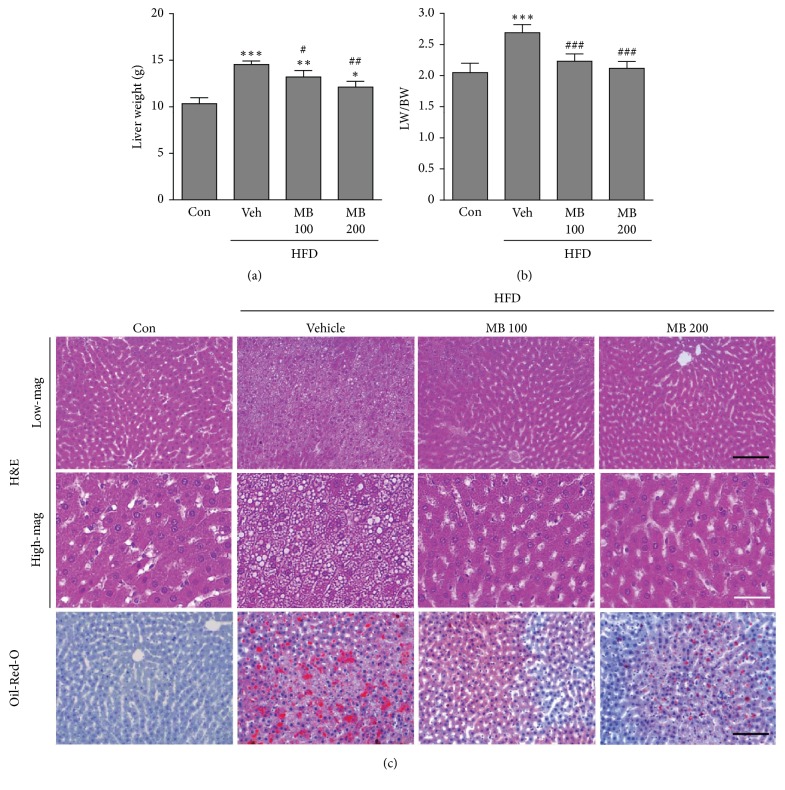
**MB prevents hepatic steatosis in HFD-induced NAFLD**. (a) Liver weight and (b) ratio of liver weight to body weight were determined in HFD-fed rats after MB treatment for 10 weeks (n=10 per group). (c) H&E and Oil-Red-O staining were performed using liver tissues in HFD-fed rats after MB treatment for 10 weeks (n=5 per group). Data are mean ± standard error of the mean (SEM). Significance was measured using one-way analysis of variance (ANOVA) followed by Bonferroni's post hoc test. ^*∗*^*P* < 0.05, ^*∗∗*^*P* < 0.01, and ^*∗∗∗*^*P* < 0.005 vs. Control group; ^#^*P* < 0.05, ^##^*P* < 0.01, and ^###^*P* < 0.001 vs. HFD-fed group. Con: control group; Veh: vehicle-treated group; MB 100 and 200, 100, and 200 mg/kg mulberry extracts-treated groups. HFD: high fat diet; LW: liver weight; BW: body weight; high-mag: high-magnification; low-mag: low magnification. Black scale bar: 50*μ*m; white scale bar: 100*μ*m.

To evaluate the effects of MB on hepatic steatosis, we examined liver morphology in HFD-induced NAFLD rats after treatment with MB for 10 weeks. Both liver weight and ratio of liver to body weight increased in HFD-fed rats compared with the control group. Treatment with MB significantly inhibited the increase in liver weight in the HFD-fed group (Figures 1(a) and 1(b)). In addition, to evaluate the effects of mulberry on hepatic steatosis, we performed histological evaluation of liver tissues stained with H&E and Oil-Red-O to visualize lipid accumulation. As shown in Figure 1(c), liver cells exhibited the severe steatosis shown by many cytoplasmic fat vacuoles and small lipid droplets in the HFD-fed group compared with the control group. However, MB treatment remarkably inhibited hepatic intracellular lipid accumulation compared with the HFD-fed group.

### 3.3. Effects of MB on Serum, Hepatic Lipid Levels, and Hepatic Damage Markers in HFD-Induced NAFLD

**Figure 2:**
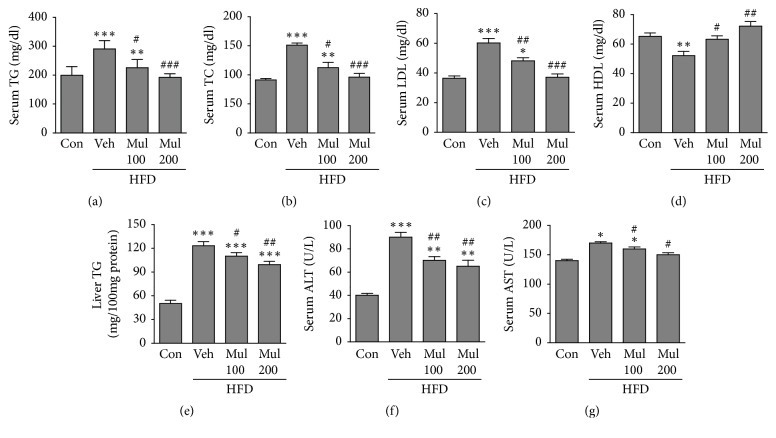
MB ameliorates serum and hepatic lipid levels and hepatic damage markers in HFD-induced NAFLD. Serum (a) TG, (b) TC, (c) LDL, and (d) HDL and (e) hepatic TG, serum (f) ALT, and (g) AST were determined in HFD-fed rats after MB treatment for 10 weeks (n=10 per group). Data are mean ± standard error of the mean (SEM). Significance was measured using one-way analysis of variance (ANOVA) followed by Bonferroni's post hoc test. ^*∗*^*P* < 0.05, ^*∗∗*^*P* < 0.01, and ^*∗∗∗*^*P* < 0.005 vs. control group; ^#^*P* < 0.05 and ^##^*P* < 0.01 vs. HFD-fed group. Con, control group; Veh, vehicle-treated group; MB 100 and 200, 100, and 200 mg/kg BW mulberry extracts-treated groups. TG: triglycerides; TC: total cholesterol; LDL: low-density lipoprotein cholesterol; HDL: high-density lipoprotein cholesterol; ALT: alanine aminotransferase; AST: aspartate aminotransferase.

To examine the effect of MB on hepatic lipotoxicity in HFD-fed rats, we tested serum and hepatic lipid parameters after treatment with MB in HFD-fed rats. As shown in Figures 2(a)–2(c), serum TG, TC, and LDL-cholesterol levels were significantly increased, but HDL-cholesterol level was decreased in the HFD group compared with the control group (Figure 2(d)). However, in HFD-fed rats treated with both 100 and 200 mg/kg BW of MB, the levels of serum TG, TC, and LDL were significantly reduced compared with the HFD-group (Figures 2(a)–2(c)). On the other hand, HDL level was significantly increased in the MB-treated HFD groups compared with the HFD group (Figure 2(d)). Hepatic TG level was also significantly reduced in the MB-treated HFD groups compared with the HFD group (Figure 2(e)). In addition, serum levels of AST and ALT as hepatic damage markers were dramatically inhibited after treatment with MB in HFD-fed rats (Figures 2(f) and 2(g)).

### 3.4. Effects of MB on mRNA Expression of Hepatic Cholesterol Homeostasis-Related Genes in HFD-Induced NAFLD

**Figure 3:**
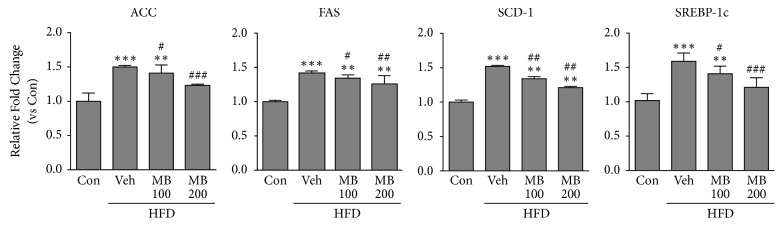
MB suppresses mRNA expression of hepatic cholesterol homeostasis-related genes. qRT-PCR analysis for ACC, FAS, SCD-1, and SREBP-1c was performed in HFD-fed rats after MB treatment for 10 weeks (n=10 per group). The qRT-PCR analysis was conducted in triplicate with five independent samples. Data are mean ± standard error of the mean (SEM). Significance was measured using one-way analysis of variance (ANOVA) followed by Bonferroni's post hoc test. ^*∗∗*^*P* < 0.01 and ^*∗∗∗*^*P* < 0.005 vs. control group; ^#^*P* < 0.05, ^##^*P* < 0.01 and ^###^*P* < 0.001 vs. HFD-fed group. Con: control group; Veh: vehicle-treated group; MB 100 and 200, 100, and 200 mg/kg mulberry extracts-treated groups. HFD, high fat diet.

To determine whether MB treatment could affect cholesterol regulatory mechanisms in the liver, mRNA expression of several hepatic cholesterol homeostasis-related genes was evaluated by qRT-PCR. The results showed that mRNA levels of cholesterol homeostasis-related genes, such as SREBP-1C, FAS, CPT-1, and SCD-1, were significantly increased in the liver tissues of the HFD-fed group compared with those in the control group. By contrast, MB treatment significantly decreased the mRNA levels of these genes compared with the HFD group (Figure 3).

### 3.5. Effects of MB on Hepatic Oxidative Stress and Mitochondrial ROS Production in HFD-Induced NAFLD

**Figure 4:**
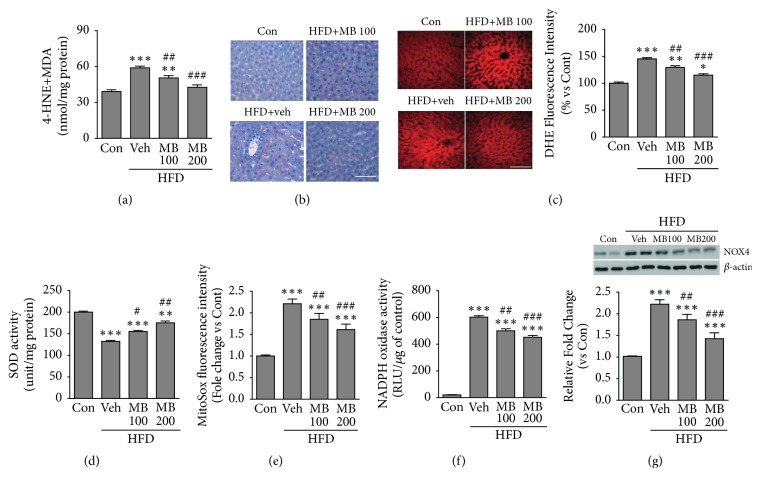
MB inhibits hepatic oxidative stress and mitochondrial ROS production in HFD-induced NAFLD. (a) 4-HNE and MDA activities were assessed using liver tissues in HFD-fed rats after Mul treatment for 10 weeks (n=10 per group). (b) 4-HNE and (c) DHE staining were performed in liver sections from HFD-fed rats after MB treatment for 10 weeks (n=10 per group), and DHE fluorescence intensity was quantified by detection of superoxide anions in liver tissues. Mitochondrial ROS production was assessed by determination of (d) SOD, (e) MitoSOX fluorescence, and (f) NADPH activities in HFD-fed rats after MB treatment for 10 weeks (n=10 per group). (g) Protein expression of NOX4 was measured by western blot analysis. The band densities were measured using NIH ImageJ software. *β*-actin was used as a loading control. Data are mean ± standard error of the mean (SEM). Significance was measured using one-way analysis of variance (ANOVA) followed by Bonferroni's post hoc test. ^*∗*^*P* < 0.05, ^*∗∗*^*P* < 0.01, and ^*∗∗∗*^*P* < 0.005 vs. control group; ^#^*P* < 0.05, ^##^*P* < 0.01, and ^###^*P* < 0.001 vs. HFD-fed group. Con: control group; Veh: vehicle-treated group; MB 100 and 200, 100, and 200 mg/kg mulberry extracts-treated groups. HFD: high fat diet. Scale bar: 50*μ*m.

Since NAFLD is associated with increased oxidative stress, including synthesis of lipid peroxidation products such as MDA and 4-HNE, we examined the preventive effect of MB against oxidative stress in HFD-fed rats. The results showed that hepatic MDA and 4-HNE proteins were considerably inhibited in the MB-treated HFD groups compared with the HFD group, while these proteins in the HDF group were significantly increased compared with the control group (Figure 4(a)). Furthermore, both 4-HNE and DHE staining demonstrated the inhibitory effects of MB against the production of MDA and 4-HNE proteins in HFD-fed rats. (Figures 4(b) and 4(c)). We next determined whether MB suppresses mitochondrial ROS production and oxidative damage in liver tissues. First, activity of hepatic superoxide dismutase (SOD), an antioxidant protein, was significantly preserved by MB treatment in HFD-fed rats (Figure 4(d)). MitoSOX assay, a mitochondrial superoxide indicator, revealed that mitochondrial ROS production was significantly inhibited by MB treatment (Figure 4(e)). Finally, because previous reports have shown that NADPH oxidase plays an important role in the pathogenesis of oxidative stress in NAFLD [34], activity and expression levels of NADPH were assessed. As expected, MB significantly decreased hepatic NADPH oxidase activity (Figure 4(f)). Western blot analysis further showed that MB significantly inhibited the HFD-induced increased level of NOX 4 protein (Figure 4(g)).

### 3.6. Effects of MB on Activities of Mitochondrial OXPHOS Complexes and ATP Contents in HFD-Induced NAFLD

**Figure 5:**
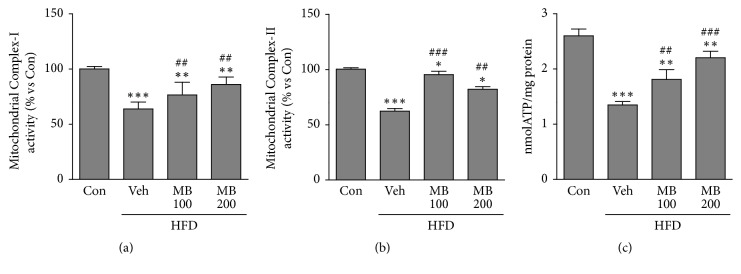
MB preserves activities of mitochondrial OXPHOS complexes and ATP contents in HFD-induced NAFLD. Activities of mitochondrial OXPHOS complexes, including (a) complex I and (b) complex II, were measured in HFD-fed rats after MB treatment for 10 weeks (n=10 per group). (c) ATP contents were luminometrically measured in HFD-fed rats after MB treatment for 10 weeks (n=10 per group). Data are mean ± standard error of the mean (SEM). Significance was measured using one-way analysis of variance (ANOVA) followed by Bonferroni's post-hoc test. ^*∗*^*P* < 0.05, ^*∗∗*^*P* < 0.01, and ^*∗∗∗*^*P* < 0.005 vs. control group; ^##^*P* < 0.01 and ^###^*P* < 0.001 vs. HFD-fed group were measured in HFD-fed rats after MB treatment for 10 weeks (n=10 per group). (c) ATP contents were luminometrically measured in HFD-fed rats after MB treatment for 10 weeks (n=10 per group). Data are mean ± standard error of the mean (SEM). Significance was measured using one-way analysis of variance (ANOVA) followed by Bonferroni's post hoc test. ^*∗*^*P* < 0.05, ^*∗∗*^*P* < 0Con: control group; Veh: vehicle-treated group; MB 100 and 200, 100, and 200 mg/kg mulberry extracts-treated groups. HFD: high fat diet.

The enzyme activities of mitochondrial OXPHOS complexes were measured after treatment with MB in HFD-fed rats. This is because oxidative stress causes OXPHOS complexes dysfunctions in mitochondria. Clear reductions in complex I and III activities were shown in the HFD group compared with the control group; these levels were significantly increased in the MB-treated HFD-fed groups (Figures 5(a) and 5(b)). In addition, hepatic ATP content was also significantly reduced in HFD-fed rats; however, these levels were dramatically rescued in MB-treated groups compared with the HFD group (Figure 5(c)).

## 4. Discussion

Previous studies have demonstrated that mulberry extract contains large amounts of flavonoids, phenolic compounds, and anthocyanins, which exhibit many bioactive features including antioxidant, anti-inflammatory, antiobesity, antiatherogenesis, and anticancer properties [29, 35–37]. Indeed, mulberry leaf extract and its active compounds show preventive effects against various diseases, including hyperlipidemia, diabetes, and tumors [20, 22, 38]. Particularly, mulberry leaf extract is reported to protect against hepatic dysfunction and NAFLD [39]. However, the pharmacological activity of mulberry fruit is less studied than that of mulberry leaf. Therefore, the present study evaluated the potential effects of mulberry fruit extract against NAFLD using the HFD-induced rats/NAFLD, which exhibits obesity and metabolic syndrome and further induces hepatic injury and morphological changes.

In this study, HFD feeding for 10 weeks caused to significant increase in serum TC, TG, and LDL-cholesterol, but a decrease in serum HDL-cholesterol. Significant reduction in these serum lipid parameters was observed after MB treatment in HFD-fed rats. MB also suppressed the hepatic injury markers including serum ALT and AST, as well as hepatic TG level as a hepatic lipid parameter. Moreover, liver steatosis, which is characterized by cytoplasmic vacuoles and small lipid droplets formation, was shown by histological analysis to be prevented by MB treatment. MB treatment further reduced the levels of cholesterol homeostasis-related genes, including SREBP1c, FAS, SCD-1, and CPT-1, in HFD-fed rats. These results indicate that MB treatment is effective for regulating dyslipidemia and liver steatosis in HFD-induced NAFLD rats.

Importantly, oxidative stress is closely associated with development and progression of NAFLD [40]. Indeed, the accumulation of hepatic lipids causes overproduction of ROS, resulting in oxidative stress in HFD-induced rats/NAFLD [41]. Lipid oxidation due to HFD is the main source of ROS production in the liver. Here, we demonstrated that MB dramatically inhibits oxidative stress resulting from the inhibition of both 4-HNE as a lipid peroxidation product and DHE as a ROS production marker in HFD-fed rats. In addition, MB also increases activity of SOD enzyme, an antioxidant protein, in NAFLD-induced rats. Next, since mitochondrial dysfunction is critical for the progression of NAFLD, mitochondrial functional studies were performed to determine whether MB could preserve mitochondrial function in HFD-induced NAFLD. Recent studies have reported that the structure and function of mitochondria are altered and further provoke metabolic disorders in HFD-induced rats/NAFLD [42, 43]. These mitochondrial dysfunctions could be linked to ROS formation, which have a pivotal role in liver damage in NAFLD of HFD-induced rats. In this study, we found that MB preserved mitochondrial functions against NAFLD by performing a MitoSOX assay. Increases in both NOX activity and protein expression of NOX4, which is localized in mitochondria and is the major source of ROS in the liver [34], are dramatically prevented by MB treatment in HFD-fed rats. We further confirmed that MB treatment also preserves the OXOPHOS complex involved in the mitochondrial bioenergetics in HFD-induced rats/NAFLD. Our results reveal that MB inhibits hepatic oxidative stress and mitochondrial-induced ROS production in HFD-induced rats/NAFLD.

## 5. Conclusions

In conclusion, our study shows that MB has the potential to ameliorate NAFLD in HFD-fed rats. Taken together, our results demonstrate that MB inhibits oxidative stress in HFD-induced rats/NAFLD for the related underlying mechanism. This study provides evidence that MB could be considered as a potential dietary supplement strategy for prevention and treatment of NAFLD.

## Figures and Tables

**Table 1:** HPLC analysis of the anthocyanic compositions in MB.

Anthocyanin	Content (mg/g)
Cyanidin-3-glucosde (C3-G)	153.7
Cyanidin-3-rutin (C3-R)	53.6

The chromatograms were monitored at 518 nm, which corresponds to anthocyanic compositions. MB: mulberry fruit extract.

## Data Availability

The data used to support the findings of this study are included within the article.
